# *Anoectochilus burmannicus* Extract Rescues Aging-Related Phenotypes in *Drosophila* Susceptible to Oxidative Stress-Induced Senescence

**DOI:** 10.3390/ijms26125694

**Published:** 2025-06-13

**Authors:** Pensiri Buacheen, Jirarat Karinchai, Woorawee Inthachat, Chutikarn Butkinaree, Ariyaphong Wongnoppavich, Arisa Imsumran, Piya Temviriyanukul, Yoshihiro H. Inoue, Pornsiri Pitchakarn

**Affiliations:** 1 Department of Biochemistry, Faculty of Medicine, Chiang Mai University, Muang Chiang Mai, Chiang Mai 50200, Thailand; pensiri.bua@cmu.ac.th (P.B.); jirarat.karin@gmail.com (J.K.); ariyaphong.w@cmu.ac.th (A.W.); arisa.bonness@cmu.ac.th (A.I.); 2 Institute of Nutrition, Mahidol University, Salaya, Nakhon Pathom 73170, Thailand; woorawee.int@mahidol.ac.th (W.I.); piya.tem@mahidol.ac.th (P.T.); 3 National Center for Genetic Engineering and Biotechnology, National Science and Technology Development Agency, 113 Thailand Science Park, Phahonyothin Road, Khlong Nueng, Khlonag Luang, Pathum Thani 12120, Thailand; chutikarn.but@biotec.or.th; 4 Biomedical Research Center, Kyoto Institute of Technology, Matsugasaki, Kyoto 6060962, Japan

**Keywords:** age-related disorder, functional food, longevity promotion, locomotor activity, medicinal plants, orchid, oxidative stress

## Abstract

Aging is a significant risk factor for various conditions, including neurodegeneration, cardiovascular disease, and type 2 diabetes. The accumulation of reactive oxygen species (ROS) and a decline in antioxidant defense are mechanisms that are widely acknowledged as causing the acceleration of both aging and the onset of age-related diseases. To promote longevity and reduce the risk of the development of aging-related disorders, it is essential to prevent or minimize oxidative stress and enhance antioxidant defense. It has been shown that *Anoectochilus burmannicus* (AB), a jewel orchid rich in phenolic compounds, can impact various biological activities associated with aging prevention. These activities include antioxidant, anti-inflammation, anti-insulin resistance, and anti-obesity effects. The aim of this study was to explore whether AB extract (ABE) could serve as an anti-aging agent using a *Sod1*-deficient *Drosophila* model, which accelerates the process of aging through ROS production. The results demonstrated that ABE, at a concentration of 2.5 mg/mL, significantly extended the lifespan of the flies and helped maintain their locomotor activity as they aged. ABE also reduced the age-related accumulation of damaged proteins in the muscle of the flies by inhibiting the expression of *Gstd1*, a genetic marker for oxidative stress. This finding agrees with those from in vitro experiments, which have shown the potential for ABE to reduce the production of ROS induced by H_2_O_2_ in myoblasts. ABE has been shown to attenuate insulin resistance, an age-related disorder, by inhibiting the pro-inflammatory cytokine TNF-α, which in turn increased insulin-stimulated glucose uptake in adipocytes. These findings suggest a promising role of ABE as an ingredient in functional foods or nutraceuticals aimed at promoting health, preventing oxidative stress, and potentially managing age-associated diseases.

## 1. Introduction

Aging is the process of becoming older, characterized by the degeneration of biological processes that leads to a progressive and irreversible decline in physical function across all organ systems. The degenerative processes also increase the risk of age-related chronic diseases, including neurodegenerative disorders, cardiovascular disease, and cancer, all of which negatively impact quality of life and can lead to increased mortality [1,2]. Various factors can accelerate the aging process, including oxidative stress, glycation, telomere shortening, side reactions, mutations, and protein aggregation [3]. The theory of aging linked to oxidative stress suggests that the functional losses associated with aging are primarily caused by the accumulation of damage induced by reactive oxygen species (ROS) [4]. Thus, the maintenance of balanced levels of ROS and the boosting of the antioxidant defense system in aging adults may be effective strategies for the prevention of age-related diseases and the improvement of the overall health status of the elderly.

Oxidative stress is defined as an imbalance between pro-oxidant and antioxidant species. An excessive accumulation of ROS can damage nucleic acids and oxidize lipids or proteins, resulting in a decline in mitochondrial function. This decline leads to further oxidative stress, which can harm cells, accelerate aging, and contribute to the development of age-related diseases [5]. Several factors associated with the generation of ROS include stress, aging, cellular metabolism, radiation, chemical agents, and both acute and chronic diseases. ROS can oxidize cellular components, particularly proteins, producing abnormal proteins that are targeted by ubiquitin for degradation via the proteasomes [6]. However, excessive protein degradation has been linked to aging and cell death, specifically through apoptosis or necrosis [7]. There is evidence to show that the efficiency of protein quality control mechanisms declines with age, resulting in the accumulation of damaged or misfolded proteins. These proteins may form aggregates, which disrupt the function of organelles and contribute to tissue dysfunction and a shorter lifespan [8,9]. Previous studies have reported a significant increase in oxidized proteins associated with age across various models, including rat hepatocytes [10], human erythrocytes [11], human brain [12], and the whole bodies of fruit flies [13]. The accumulation of oxidatively damaged proteins has also been observed in age-related diseases such as Alzheimer’s and Parkinson’s, which are associated with neurodegenerative disorders, as well as in sarcopenia, the degenerative loss of skeletal muscle mass [14].

The prevalence of insulin resistance is on the rise among the elderly population [15]. Age-related changes play a significant role in this increase, particularly with regard to alterations in the distribution of adipose tissue, specifically adipogenesis, the development of features associated with brown adipose tissue, and changes in adipokine release. These characteristics are linked to both inflammatory conditions and lipotoxicity. Finally, they contribute to inflammation-induced insulin resistance in adipose tissue, which is a key factor in the development of type 2 diabetes [16,17]. Thus, the reduction of inflammation-induced insulin resistance in adipose tissue during aging could be an effective strategy for the prevention of type 2 diabetes and the promotion of healthy aging in the elderly.

The study of aging and age-related chronic disorders in humans often involves the use of animal models such as mice or rats, due to their genetic similarities to humans and parallels in organ function. However, these studies can be time-consuming, labor-intensive, and expensive. An alternative is to utilize *Drosophila melanogaster*, which has been proven to be a highly effective organism for the study of aging-related diseases and anti-aging compounds due to its short life span and rapid reproductive cycle. Approximately 60% of the *Drosophila* genome is homologous to that of humans, and about 75% of genes associated with human diseases have counterparts in flies [18]. Aged *Drosophila* exhibit impaired locomotor activity and age-related phenotypes, including the accumulation of abnormal protein aggregates containing polyubiquitinated proteins in their muscles and the loss of dopaminergic (DA) neurons in their brains [19,20]. With regard to the oxidative stress theory of aging, the superoxide dismutase (SOD) enzyme has been of particular interest [21]. For instance, a hypomorphic *Sod1* mutant exhibits a redox imbalance that accelerates mortality. Moreover, *Sod1*-depleted flies display age-dependent phenotypes at an earlier stage [22]. Therefore, *Sod* mutants and tissue-specific depletion of *Sod* genes provide convenient models for the rapid screening and investigation of anti-aging compounds [22,23,24].

Many plant extracts and natural compounds possess potential antioxidant properties, either by direct scavenging of free radicals or by enhancing the body’s antioxidant defense system. For example, Le et al. reported that sesamin improved the antioxidant defense in *Drosophila* hypomorphic mutant flies with excessive ROS accumulation. This was achieved by an increase in the expression of antioxidant enzymes, including catalase and superoxide dismutase (SOD) 1 and SOD2 [22]. These mutant flies accumulate ROS earlier than control flies, making them highly sensitive to redox imbalance [22,25]. This *Drosophila* model has become crucial for the identification of several antioxidants with anti-aging activities that extend mean lifespan through improved antioxidant capacity [22,23,24,26,27].

The genus *Anoectochilus* belongs to the *Orchidaceae* (orchid family). Over forty *Anoectochilus* species can be found in regions such as China, India, South and Southeast Asia, Australia, and the islands in the southwest Pacific [28,29]. These plants are known as medicinal orchids and have been used in traditional medicine for various therapeutic purposes [30,31,32,33,34,35,36]. They are employed to treat a wide range of disorders, including inflammation, diabetes, menstrual problems, piles, boils, arthritis, stomach problems, and chest pain [30,32,33,35]. Specifically, *A. formasanus* and *A. roxburghii*, which contain a high content of kinsenoside, a key bioactive compound [37], have been used to cure various illnesses, including chest pain, stomachache, diabetes, hypertension, liver dysfunction, spleen dysfunction, lung disease, and tumors [30,32,34,38]. Studies have shown that these orchids exhibit antioxidant, hepatoprotection, anti-glycemic, and antihyperlipidemic activities in animal models with diabetes [39,40,41,42].

This study is focusing on *A. burmannicus* (AB) Rolfe, also known as Nok-Kum-Fai in Thai. (The plant name has been verified with WFO (https://www.worldfloraonline.org/taxon/wfo-0000216431, accessed on 11 November 2024). This orchid is native to the tropical rainforests of Southeast Asia, including Thailand, and can also be found in countries such as Bhutan, Laos, China, Vietnam, and Indonesia [43]. *A. burmannicus* is characterized by its succulent and relatively weak structure and oval-shaped leaves with a dark purple color and netted or reticulated venation. The collection of *A. burmannicus* is legally regulated in some countries, including Thailand, where its habitats are protected as conservation areas. *Anoectochilus* species can be efficiently propagated through tissue culture techniques, which allow for the rapid and large-scale production of disease-free plants of consistent quality, leading to the feasibility of the commercial use of AB. Previous research by Budluang et al. found that an aqueous extract of AB exhibits antioxidant, anti-inflammatory, and anti-insulin resistance activities [43]. Similarly, Karinchai et al. reported that the AB ethanolic extract (ABE) also exhibits antioxidant and anti-inflammatory activities [44]. These studies suggest that both kinsenoside and other uncharacterized compounds within ABE act as potential bioactive molecules. There is also evidence to indicate that ABE and kinsenoside may act as anti-obesity agents by inhibiting lipid accumulation induced by obesogens in 3T3-L1 adipocytes [45]. Given the antioxidant and anti-inflammatory properties of this plant, coupled with its high phenolic content and presence of kinsenoside, the rationale for carrying out this study is that ABE may help slow down aging processes linked to oxidative stress. This beneficial effect might result either from the modulation of the production of oxidative stress and the enhancement of defensive responses or the inhibition of inflammation and its associated pathogenesis.

The aim of this study is to determine the anti-aging properties of ABE through oxidative suppression using genetically modified *Drosophila* as an accelerated in vivo aging model. The effect of ABE on the life span of *Drosophila* mutants lacking the *Sod1* gene, a model known for its redox imbalance and shorter life span, was initially investigated. The effect of ABE on key aging phenotypes, including locomotor activity and the accumulation of abnormal protein aggregation in muscle, was then investigated further in muscle-specific *Sod1* depletion flies. To monitor the influence of ABE on the accumulation of oxidative stress, the level of expression of the oxidative stress-responsive gene, *Gstd1,* in flies was analyzed. Finally, the potential for ABE to slow aging via its antioxidant and anti-inflammation activities was explored by investigating its effect on intracellular ROS production in C2C12 myoblasts and on inflammation-induced insulin resistance in 3T3-L1 adipocytes.

## 2. Results

### 2.1. The Effect of Anoctochillus burmannicus Ethanolic Extract (ABE) on the Lifespan Extension and the Locomotor Activity Improvement in a Drosophila Aging Model

**Figure 1:**
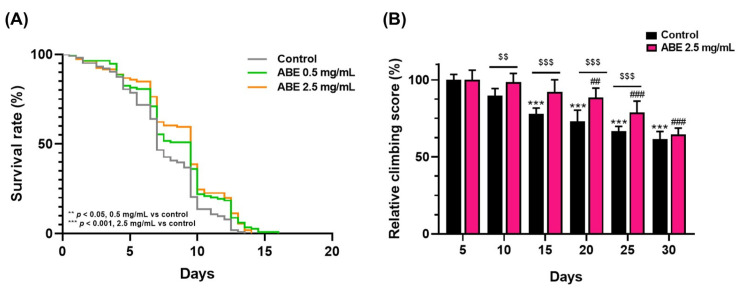
The effect of ABE consumption on adult life span (**A**) and locomotor activity (**B**) in a Drosophila aging-accelerated model. (**A**) Survival curve plotted by Kaplan–Meier survival analysis of *Sod1^n1^* mutant flies fed with or without ABE (control, n = 103; 0.5 mg/mL ABE, n = 114; 2.5 mg/mL ABE, n = 106). ** *p* < 0.05, *** *p* < 0.001 compared to control, Log-rank test. (**B**) The effect of ABE (2.5 mg/mL) on locomotor activity of muscle-specific Sod1 depletion in Drosophila adults. The climbing score over time is shown as relative to the day 5 value. Data are represented as mean ± SD (n = 100). One-way ANOVA with Tukey’s multiple comparisons test, *** *p* < 0.001 compared to day 5 value (control group), ## *p* < 0.05, ### *p* < 0.001 compared to day 5 value (ABE group). $$ *p* < 0.05, $$$ *p* < 0.001 control group compared to ABE-treated group.

One of the primary causes of aging is oxidative stress, which occurs when there is an imbalance between antioxidant defense and free radical activity. Lifespan measurement is a key approach employed to evaluate the effects of genetic and non-genetic factors on the aging process. In an initial investigation into the impact of ABE on the lifespan of *Drosophila*, we employed *Sod1^n1^* mutant flies. Higher levels of ROS accumulate in these mutants due to defects in their redox balance systems. The results showed that these mutant flies had a significantly shortened adult lifespan, with a 50% survival rate of only about 7 days (Figure 1A). Interestingly, when these mutant flies were fed with ABE at 0.5 and 2.5 mg/mL, their 50% survival rate increased significantly to 9.5 days (*p* < 0.05 and *p* < 0.001, respectively). These results suggest that ABE has the potential to delay accelerated aging and reduce the mortality rate in adult flies.

Aging is associated with a gradual decline in locomotor activities, making it essential to maintain locomotor ability for a good quality of life. We further investigated whether the consumption of ABE can improve age-related impairment in locomotor activity. The effect of ABE on climbing activity was assessed in adult *Drosophila* with muscle-specific *Sod1* depletion. On day 15 of the experiment, the relative climbing activity of each treatment group compared to the control group was measured. It was found that the group treated with 2.5 mg/mL of ABE showed a trend toward the preservation of climbing activity until experimental day 25. This was shown by a significantly higher climbing score in comparison to the control group (Figure 1B). In addition, the relative climbing score of the ABE group was significantly higher than that of the control group from day 10 until day 25. These results suggest that a high dose of ABE may help maintain locomotor functions in aging *Drosophila*. Taken together, ABE exerted anti-aging activity by extending lifespan and preserving locomotor activity in flies that experience accelerated aging.

### 2.2. The Suppressive Effect of ABE on Age-Dependent Accumulation of Abnormal Protein Aggregates and Expression of a Marker Gene Induced by Oxidative Stress in Drosophila Adult Muscle

**Figure 2:**
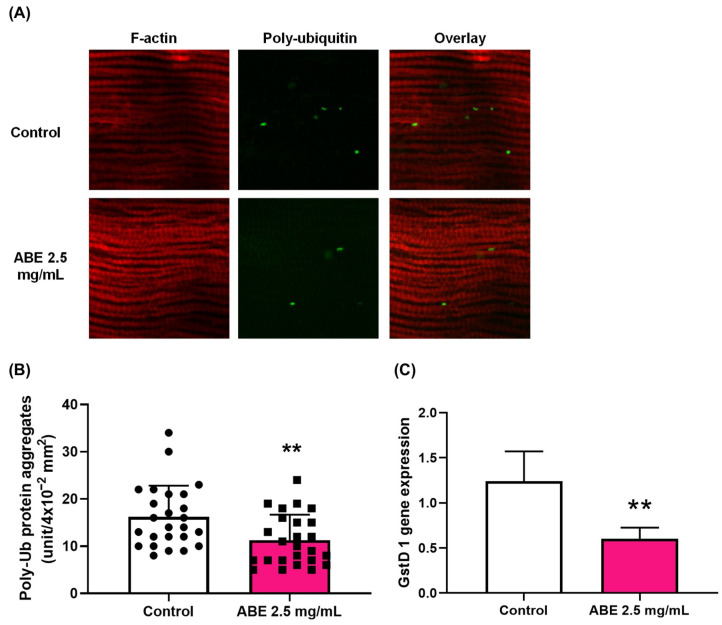
The effect of ABE on protein aggregation in indirect flight muscle (IFM) (**A**,**B**) and expression of the oxidative response gene, *GstD1*, (**C**) in *Drosophila* adults. (**A**) Immunostaining of the indirect flight muscles from the model flies with a muscle-specific depletion of *Sod1* (*Mef2 > Sod1RNAi*) using an anti-ubiquitin-conjugated antibody (green) and phalloidin staining for F-actin (red). (**A**) Flies were fed with a control diet or with an ABE-supplemented diet (ABE 2.5 mg/mL) for 12 days after eclosion. (**B**) The average number of protein aggregates containing polyubiquitinated proteins in each confocal optic field. The area of ubiquitinated protein aggregates (green) was analyzed using ImageJ software (version 1.54g) and converted into the number of protein aggregates as a unit/4 × 10^−2^ mm^2^. Circles and squares represent the number of protein aggregates in each muscle of the control group (n = 25) and the ABE feeding group (n = 25), respectively. (**C**) Quantitation of mRNA of *GstD1*, a marker gene used to monitor the oxidative stress accumulation in the flies (Mef2 > Sod1RNAi) fed with control diet or ABE-containing diet. Data are represented as mean ± SD from two independent experiments (n = 10, total n = 20). Student’s *t*-test ** *p* < 0.01.

The accumulation of damaged or misfolded proteins, which form protein aggregates, is a result of the declining effectiveness of protein quality control mechanisms during aging. These aggregated proteins can disrupt the function of organelles, leading to tissue dysfunction and a shorter lifespan. As the above results indicate that ABE (2.5 mg/mL) improves both the lifespan and locomotor activity in aged flies, the effect of ABE on the accumulation of polyubiquitinated (poly-Ub) protein aggregates in the muscles of aged flies was further investigated. Figure 2A shows the poly-Ub protein aggregates in muscle as green spots. The area of the green spots was converted into the number of protein aggregates, as shown in Figure 2B. The results revealed that ABE (2.5 mg/mL) significantly decreased the levels of poly-Ub protein aggregates compared to the control group, as shown in Figure 2A,B. This finding suggests that ABE may improve lifespan and locomotor activity by suppressing the process of abnormal protein aggregation in the muscles of aged flies.

In the free radical theory of aging, the accumulation of ROS can cause protein damage and, in turn, abnormal protein aggregation in several organs [46]. Since it has been reported that ABE exhibits scavenging activity [44], it was postulated that ABE reduces the accumulation of abnormal protein aggregates in muscle by decreasing ROS levels. To test this hypothesis, the expression of a gene that serves as a marker for ROS accumulation after ABE consumption was measured. The gene *Gstd1*, which encodes for glutathione S transferase D1, is known to increase with age [47]. Therefore, *Gstd1* expression in response to oxidative stress can be employed as an indicator of oxidative stress [47]. Our results showed that treatment with ABE (2.5 mg/mL) significantly decreased the expression level of *Gstd1* mRNA compared to the control group (Figure 2C), suggesting that ABE may reduce the accumulation of ROS in muscle tissues, particularly in Sod1-depleted flies. Taken together, it appears that ABE can suppress the aggregation of abnormal proteins in muscle by decreasing ROS production, which in turn may lead to improvements in locomotor activity and lifespan in aging-accelerated *Drosophila*.

### 2.3. Cytotoxicity of ABE on C2C12 Myoblasts and 3T3-L1 Adipocytes

**Figure 3:**
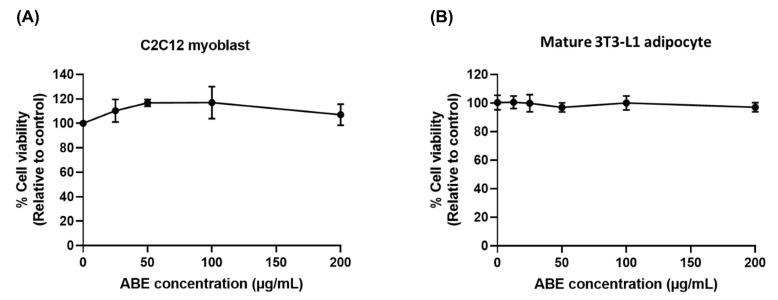
Cytotoxicity of ABE on (**A**) C2C12 myoblasts and (**B**) mature 3T3-L1 adipocytes. The cells were treated with various doses of ABE (12.5–200 µg/mL) for 48 h. Cell viability was determined by sulforhodamine B (SRB) assay. The data are shown as mean ± SD of three independent experiments.

To investigate the inhibitory effect of ABE on ROS production in C2C12 myoblasts and its effect on insulin resistance in 3T3-L1 adipocytes, non-toxic concentrations of ABE were used in both cell types. As shown in Figure 3, the cell viability of both cell types after ABE treatment was not significantly different from the control, indicating that ABE was non-toxic to C2C12 myoblasts and mature 3T3-L1 adipocytes at concentrations up to 200 µg/mL. Therefore, non-toxic concentrations ranging from 50 to 200 µg/mL were selected for further experiments.

### 2.4. The Inhibitory Effect of ABE on Cellular ROS Production Induced by H_2_O_2_ in C2C12 Myoblasts

**Figure 4:**
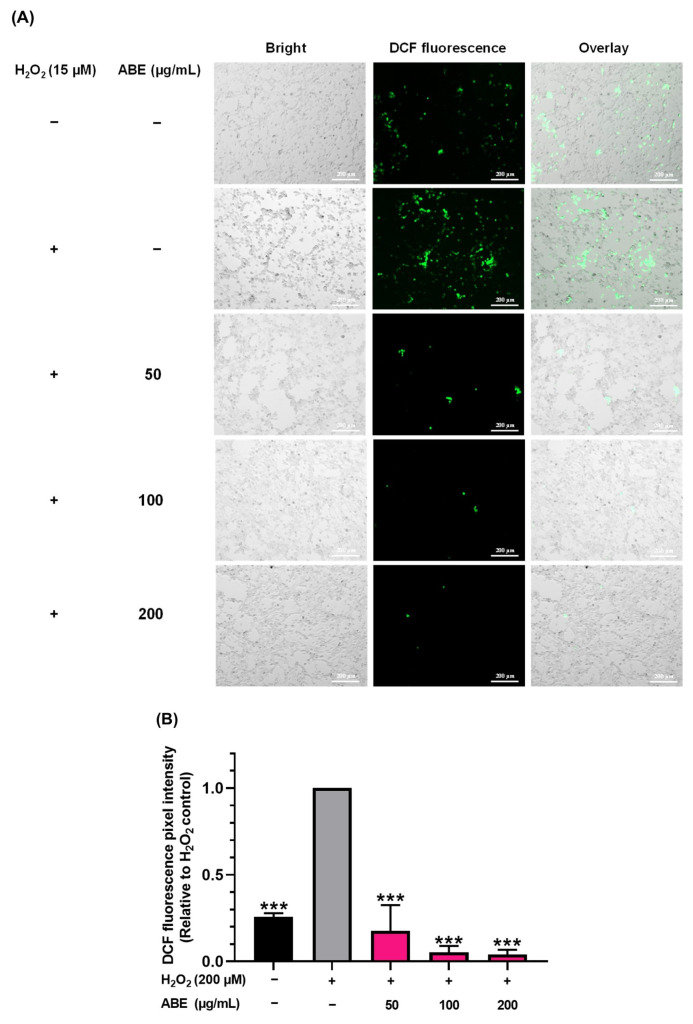
The effect of ABE against H_2_O_2_-generated intracellular ROS in C2C12 myoblasts. (**A**) Fluorescent microscopy images of the cells, a bright-field image showing the general morphology of the cells (left panel), a fluorescent image showing the DCF fluorescence (middle panel), and an overlay image combining bright-field and fluorescent images to illustrate the spatial relationship between cellular structures and fluorescent signals (right panel). (**B**) Relative DCF fluorescence pixel intensity as analyzed by the Zeiss ZEN Pro software (version 3.9). Data are represented as mean ± SD of triplicate experiments and analyzed using a one-way ANOVA with Tukey’s multiple comparisons test. *** *p* < 0.001 compared to H_2_O_2_-treated controls.

To gain a deeper understanding of the antioxidant potential of ABE against oxidative stress-induced aging, the impact of ABE on the reduction of the production of ROS in C2C12 myoblasts using the DCFDA assay was investigated. As shown in Figure 4A,B, treatment with H_2_O_2_ significantly increased intracellular ROS levels, as indicated by the enhanced intensity of DCF fluorescence in comparison to the untreated control. However, DCF fluorescence was significantly reduced in cells pretreated with 50–200 µg/mL of ABE, at approximately 91–97% inhibition compared to the H_2_O_2_-treated controls. Interestingly, the DCF fluorescence intensity in the ABE-treated cells was significantly lower than that of the untreated control group, suggesting that ABE may also be effective in the elimination of endogenous ROS by enhancing the intracellular antioxidant system. These results confirm the cellular antioxidant activity of ABE as a result of its role in reducing ROS production.

### 2.5. The Protective Effect of ABE on Inflammation-Induced Insulin Resistance in TNF-α Treated 3T3-L1 Adipocytes

**Figure 5:**
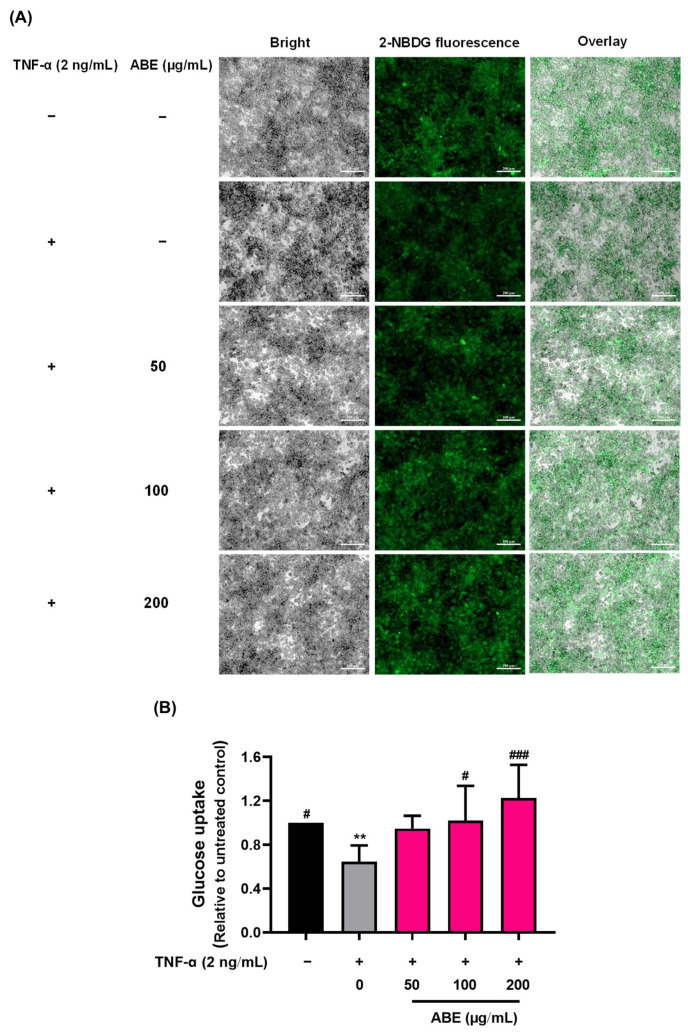
The effect of ABE on insulin-induced cellular glucose uptake in TNF-α-treated 3T3-L1 adipocytes. (**A**) Fluorescent microscopic images of the cell, the bright-field image showing the general morphology of the cells (left panel), a fluorescent image showing the 2-NBDG fluorescence (middle panel), and overlay image combining bright-field and fluorescent images to illustrate the spatial relationship between cellular structures and fluorescent signals (right panel). (**B**) Cellular uptake of 2-NBDG measured by a fluorescence microplate reader. Data presented as mean ± SD of triplicate experiments and analyzed with a one-way ANOVA with Tukey’s multiple comparisons test. ** *p* < 0.01 compared to untreated controls, # *p* <0.05 ### *p* < 0.001 compared to TNF-α-treated controls.

The effect of ABE on inflammation-induced insulin resistance was determined by measuring glucose uptake in mature 3T3-L1 adipocytes. As shown in Figure 5A,B, the uptake of the glucose analog 2-NBDG decreased by approximately 35% in TNF-α-treated 3T3-L1 cells compared to the untreated controls, indicating the presence of insulin resistance. In contrast, treatment with ABE at concentrations of 50–200 µg/mL significantly increased glucose analog uptake by approximately 60–87% in comparison to the TNF-α-treated controls. These results suggest that ABE treatment could reduce inflammation-induced insulin resistance and improve insulin sensitivity in adipocytes.

## 3. Discussion

Aging is an inevitable process that involves a gradual decline in the function of tissues and organs. This process is closely linked to the development of various age-related diseases, including cardiovascular disease, type 2 diabetes mellitus, neurodegenerative diseases, sarcopenia, and cancer. As a result, there is extensive research worldwide to investigate how to slow down the aging process and delay the onset of age-related diseases to promote longevity and healthy aging. The focus of this study was to investigate the potential action of *Anoectholicus burmannicus* extract (ABE) against oxidative stress-induced aging using *Drosophila melanogaster* as a model. The effects of ABE on intracellular ROS and inflammation-induced insulin resistance using cell culture models of C2C12 myoblasts and mature 3T3-L1 adipocytes, respectively, were investigated.

The oxidative stress theory of aging suggests that the gradual accumulation of ROS causes oxidative damage to key macromolecules such as DNA, proteins, and lipids. This process, combined with a decline in the antioxidant defense system, contributes to a decrease in physiological functions and a shorter life expectancy [4,48]. ROS can arise from both endogenous sources (e.g., the electron transport chain in mitochondria and peroxisomal beta-oxidation) and exogenous sources (environmental factors, e.g., cigarette smoke, alcohol, and ionizing and UV radiation). Previous studies have demonstrated that ROS production is significantly elevated in various tissues of aged mice, including the brain [49], heart [50], skeletal muscle [51], and bone marrow hematopoietic stem cells [52]. Furthermore, antioxidant activity in older individuals is significantly lower compared to younger individuals, resulting in high levels of ROS that accelerate the aging process and shorten lifespan [53,54]. Superoxide dismutase (SOD) is a key detoxifying enzyme that converts harmful superoxide radicals into the less toxic hydrogen peroxide [55]. Animals deficient in SOD exhibit increased oxidative damage and more features of premature aging [25,56,57,58]. For example, SOD1 knockout mice have been shown to have approximately a 30% shorter lifespan than their control counterparts [59]. Conversely, overexpression of SOD enzymes (e.g., SOD1 or SOD2) has been linked to an increased lifespan in some animal models [48]. Therefore, improving the redox balance by increasing levels of endogenous and exogenous antioxidants to scavenge ROS may be an effective strategy for delaying the onset of age-associated diseases and extending life expectancy.

Several in vitro studies suggest that plant extracts rich in antioxidant compounds such as polyphenols, phytosterols, vitamins, and minerals may reduce the incidence of age-related disorders [60]. *Anoectochilus burmannicus* (AB), an orchid with high levels of phenolic compounds, including catechin, mangiferin, rutin, ferulic acid, luteolin, and apigenin, also contains kinsenoside [61]. It has been shown to exert various biological activities such as antioxidant, anti-inflammatory, anti-insulin resistance, and anti-obesity effects [43,44,45]. *Sod1* mutant flies, which carry a point mutation in the *Sod1* gene that results in an unstable form of the cytosolic SOD enzyme, eventually leading to rapid degradation [62,63] were employed to determine whether antioxidant and anti-inflammation properties of ABE could help to delay the progression of aging in a senescence-related model of *Drosophila*. Cytosolic SOD1, one of three SOD isoforms, plays a vital role in cellular redox processes. The *Sod1* mutant flies had higher levels of ROS, significantly shorter lifespans, and higher mortality rates within 30 days compared to the wild-type strains [22,64]. In this study, it was demonstrated that ABE could delay the mortality rate of *Sod1* mutant flies compared to the untreated controls, implying that ABE may extend life expectancy by directly scavenging ROS or boosting antioxidant defense mechanisms to combat oxidative stress. Similarly, Yang et al. reported that Chinese mugwort (*Artemisia argyi*) extends the lifespan of *Drosophila* by increasing antioxidant enzyme levels and decreasing oxidative stress [65]. In addition to a shortened lifespan, aging is typically accompanied by a decline in physical activity. The decrease in locomotor activity in aging is associated with functional impairments of the nervous or musculoskeletal systems, which can significantly impact the quality of life in the elderly.

*Drosophila* studies provide a robust platform for the examination of age-related changes, healthy aging, and the acceleration of aging due to unfavorable environmental circumstances [66]. The decline in climbing activity is easily observable in aged flies (aged 5–45 days) [67,68]. Changes in the nervous system and musculature associated with aging correlate with decreased dopamine levels in the brain of aged flies. Dopamine is an essential neuromodulator in the mammalian central nervous system for regulating attention, movement control, motivation, and cognition. A reduction in dopamine levels leads to impairment in negative geotaxis and climbing ability, indicating locomotor deficits [69,70]. The loss of dopaminergic neurons is related to increased oxidative damage in the brain due to aging. Increased endogenous ROS levels in the brain have been associated with decreased basal dopamine levels [71]. Moreover, deficiencies in key antioxidants, such as SOD1 and SOD2, exacerbate age-related locomotor impairments [72,73], suggesting that oxidative stress plays a significant role in the decline of locomotor function associated with aging. Muscle tissue also directly influences locomotor activity in *Drosophila*. Excessive ROS in muscle, along with the impaired ubiquitin proteasome pathway that occurs during aging, causes protein damage and the accumulation of dysfunctional proteins, which would contribute to apoptosis and cell death [6,19]. The progressive accumulation of abnormal protein aggregates in muscle can indicate an age-related phenotype in *Drosophila* [74]. Oka et al. found that depleting *Sod1* or *Sod2* in muscle is associated with increased age- and oxidative stress-related locomotor impairments. They also found an accumulation of abnormal proteins, including poly-ubiquitin, in aging fruit flies [25]. In another study, Le et al. reported that the antioxidant sesamin suppresses aging in *Drosophila* by improving the decline in locomotor activity and reducing abnormal protein aggregation in fruit flies with muscle-specific *Sod1*-depletion [22]. Similar to those findings, this study into treatment with ABE (2.5 mg/mL) indicated that the treatment tended to lead to the retention of locomotor activity in flies during the 15–25 days following eclosion, a period that corresponds to middle age in humans. Significantly, ABE also resulted in the reduction of the accumulation of ubiquitinated protein aggregates in these flies. These findings suggest that ABE may exert anti-aging effects by inhibiting the formation of abnormal protein aggregates in muscle. This action could lead to improved recovery of locomotor activity and increased longevity. Then, the effect of ABE on *Gstd1* expression, a marker gene that responds to oxidative stress and is upregulated during aging [45,75], was examined to confirm whether the mechanisms underlying the effects of ABE involve the removal of oxidative stress. The feeding of adult *Drosophila* with ABE (2.5 mg/mL) significantly decreased the level of expression of the *Gstd1* gene, suggesting that the accumulation of ROS in the flies was suppressed by this plant extract. While *Gstd1* serves as a key marker of oxidative stress in this model, future studies will expand on these findings by quantifying additional redox regulators, including superoxide dismutase (SOD), catalase (CAT), and glutathione peroxidase (GPx), to further dissect mechanistic pathways. This will provide a more comprehensive understanding of oxidative stress dynamics in the system. Taken together, it can be implied that ABE may help maintain protein homeostasis by reducing excess oxidative stress in an aging-accelerated *Drosophila* model.

In vitro experiments using H_2_O_2_-induced intracellular ROS in myoblasts ensured the antioxidant effect of ABE. It is possible that ABE enhances the activity of antioxidant enzymes, including superoxide dismutase, catalase, glutathione peroxidase, and transferase, which are essential for the control of redox balance [76]. Taken together, ABE may exhibit anti-aging effects by decreasing ROS accumulation, which in turn reduces the aggregation of abnormal proteins in muscle. This can lead to improved locomotor activity and an extended lifespan in aging *Drosophila*.

As age-related diseases significantly influence the risk of morbidity and mortality, preventing the progression of aging and its related conditions could improve the quality of life for older individuals. Among these age-related issues, insulin resistance, which develops along with type 2 diabetes, is highly prevalent in the elderly. The dysfunction of adipose tissue plays an important role in maintaining energy and metabolic balance, and its impairment with age promotes low-grade chronic inflammation, leading to insulin resistance in the elderly [77]. In addition, the accumulation of oxidative stress in tissues can inhibit adipogenesis and contribute to age-related inflammation and adipose tissue dysfunction [78]. Houstis et al. showed that ROS production induces insulin resistance in adipocytes, while ROS scavenging improves insulin sensitivity, indicating that ROS is a major contributory factor in insulin resistance [79]. Furthermore, elevated levels of ROS in adipose tissue during aging produce pro-inflammatory mediators that inhibit insulin signaling, eventually resulting in decreased insulin-stimulated glucose uptake [77,80,81]. Pro-inflammatory cytokines, such as interleukin-6 (IL-6), monocyte chemoattractant protein-1 (MCP-1), and tumor necrosis factor-alpha (TNF-α), increase in correlation with age in adipocytes [82]. Reducing the production of these inflammatory cytokines (TNF-α, IL-6, and MCP-1) has been shown to improve insulin function and stimulate glucose uptake in adipocytes [83,84]. Therefore, one effective strategy for improving health and quality of life in the elderly is to reduce inflammation-induced insulin resistance in adipose tissue as they age. Our research clearly shows that ABE significantly enhances cellular glucose uptake, suggesting that ABE has the potential to inhibit inflammation-induced insulin resistance in adipose tissue.

## 4. Materials and Methods

### 4.1. Chemicals and Reagents

Instant Drosophila Medium (Formula 4-24^®^) was obtained from Carolina Biological Supply Company (Burlington, NC, USA). Dulbecco’s Modified Eagle Medium (DMEM), fetal calf serum (FCS), penicillin, and 2 [N-(7-nitrobenz-2-oxa-1,3-diazol-4-yl0-amono) (2-NBDG) were acquired from Invitrogen (Carlsbad, CA, USA). 3-isobutyl-1-methylxanthine (IBMX), dexamethasone, insulin, and 2′,7′- dichlorofluorescin diacetate (DCFDA) were obtained from Sigma-Aldrich (St. Louis, MO, USA). Fetal bovine serum (FBS) was purchased from Hyclone Laboratories Inc. (Logan, UT, USA). TNF-α was acquired from Fisher Scientific (Leicestershire, UK). PrimeScript II Fidelity RT-PCR Kit was obtained from TaKaRa Bio Co. (Kusatsu, Shiga, Japan). The FastStart Essential DNA Green Master was bought from Roche (Basel, Switzerland). Polyubiquitinated protein was purchased from FK2, EnzoLife Sciences (Farmingdale, NY, USA). Alexa Fluor 488-conjugated secondary antibodies and rhodamine-phalloidin were obtained from Molecular Probes (Eugene, OR, USA). Vectashield was bought from Vector Laboratories (Burlingame, CA, USA).

### 4.2. Preparation of Anoctochillus burmannicus Ethanolic Extract

An *A. burmannicus* (AB) sample was tissue-cultivated and obtained from the Queen Sirikit Botanic Garden, Chiang Mai, Thailand. The herbarium voucher specimen is S. Watthana 4494 (QBG). The ethanolic extract (ABE) was prepared as previously described [44]. Briefly, the whole plant samples were washed and dried in a hot-air oven at 60 °C for 24 h. The AB plant was ground, and then the product underwent extraction using 80% ethanol in an aqueous solution at a ratio of 10:100 (*w*/*v*). The ethanolic fraction was filtered, and the solvent was removed by evaporation. Next, the extract was lyophilized to obtain crude ABE powder, yielding approximately 25% (*w*/*w*). To assess the quality of the plant sample and the extraction process, the total phenolic and kinsenoside contents were analyzed. The methods and results for these analyses have been described in our previous study [61]. In brief, the total phenolic content of ABE was found to be 11.18 ± 0.74 mg GAE/g extract and 23.28 ± 1.09 mg FAE/g extract. The kinsenoside content was 371 ± 36.75 µg/mg extract.

### 4.3. Drosophila Melanogaster Stock

*Sod1^n1^* (BDSC99584) mutant flies, *UAS-Sod1RNAi^F103^* (BDSC24493) [25] *Mef2-Gal4^ts^* (BDSC67063) [24] were obtained from the Bloomington *Drosophila* Stock Center (Bloomington, IN, USA). All *Drosophila* stocks were maintained at a constant temperature of 25 °C, on standard cornmeal molasses food, in a 12 h day/night cycle as previously described [22]. In brief, the standard cornmeal food (1 L) included cornmeal (40 g), agar (7.2 g), glucose (100 g), and dried yeast (40 g). These ingredients were mixed thoroughly and boiled for 10 min. After cooling the mixture to 70 °C, 10% parahydroxybenzonate dissolved in ethanol (5 mL) and propionic acid (5 mL) was added as a preservative. The fly food was changed every three days. The *Drosophila* study was approved by the Institute of Nutrition, Mahidol University Institutional Animal Care and Use Committee (INMU-IACUC) (COA. No. INMU-IACUC, 2024/04).

### 4.4. Lifespan Assay

The homozygous *Sod1^n1^* flies, which exhibit redox imbalance leading to a shorter lifespan, were utilized in this study. A lifespan assay was performed following established protocols [24]. In brief, flies were collected within 24 h after eclosion, and only male flies were selected to prevent accidental deaths of females during oviposition [22,24,84]. A total of 100 male flies selected at random were used for each treatment. Twenty flies were placed in each vial. The flies were raised on instant *Drosophila* Medium (Formula 4-24^®^, [24]) with or without ABE. As ABE at up to 4 mg/mL was non-toxic to the flies [44], an extract at 0.5 and 2.5 mg/mL was used for the treatment. Also, 0.5% DMSO was used as a vehicle control. The number of deceased flies was scored every 12 h, and the flies were transferred to a new diet every 3 days.

### 4.5. Climbing Assay

To generate muscle-specific *Sod1* depletion (*Mef2 > Sod1RNAi*) in flies, UAS-*Sod1RNAi^F103^* flies were crossed with *Mef2-Gal4^ts^* flies. A climbing assay was conducted as previously described [22]. Male F1 progeny with muscle-specific depletion of *Sod1* (*Mef2ts > Sod1RNAi*) were collected within 24 h after eclosion. A total of 100 flies were used for each treatment group, and twenty flies were placed at random in each vial. The flies were raised on instant food with or without ABE (0.5 or 2.5 mg/mL). 0.5% DMSO was used as a vehicle control. The food was changed every 2–3 days. Climbing activity was assessed every five days (from 5 to 45 days post-eclosion). The flies were gently taped to the bottom (10 cm in height). After 6 s, the number of flies that climbed to the marked point was counted and scored as follows: 10 points for climbing over 5 cm, 5 points for climbing below 5 cm, and 0 points for remaining at the bottom [22,85]. This process was repeated three times, and the average of the scores was calculated. The mean score from five trials (a total of 100 flies) was then calculated.

### 4.6. Ubiquitinated Protein Aggregates Accumulation in the Muscle of the Adults

Muscle-specific *Sod1* depletion (*Mef2 > Sod1RNAi*) in flies was conducted as described in previous studies [22,24,25]. The flies were collected within 24 h after eclosion, and only males were selected to eliminate sex differences. The flies were raised on instant food, with or without the addition of 0.5 or 2.5 mg/mL ABE. 0.5% DMSO was used as a vehicle control. The food was replaced every 2–3 days. After 12 days of feeding, the flies were anesthetized, and their thoraxes were dissected to collect indirect flight muscles. The muscle samples were then analyzed for ubiquitinated protein accumulation as described in previous reports [22]. In brief, the samples were fixed in 4% paraformaldehyde for 30 min. After washing with phosphate-buffered saline containing Tween 20 (PBST), the samples were blocked with 10% normal goat serum. Then, the muscle samples were incubated overnight at 4 °C with a primary mouse antibody against polyubiquitinated proteins (FK2, EnzoLife Sciences, Farmingdale, NY, USA; 1:300). After washing again with PBST, the samples were incubated with Alexa Fluor 488-conjugated secondary antibodies and rhodamine-phalloidin (Molecular Probes, Eugene, OR, USA; 1:80) to label myofibrils. This took place at room temperature for 2 h, followed by washing with PBST. Finally, the samples were mounted in Vectashield (Vector Laboratories, Burlingame, CA, USA). One image per fly was captured, and the pixel area in one optic field (4.0 × 10^−2^ mm^2^) was measured using an Olympus laser scanning confocal microscope (Fv10i, Olympus, Tokyo, Japan). The images were analyzed using ImageJ software (version 1.54g).

### 4.7. Oxidative Response Gene Expression by RT-qPCR

The treatment procedure involving flies has been previously described in Section 2.5. After a continuous feeding period of ABE for 12 days, the flies were anesthetized and their thoraxes were collected to analyze the expression of *Gstd1*, an oxidative response gene, as described by Le et al. [22]. To isolate total RNA, the thoraxes were homogenized with TRIZol reagent (Invitrogen). cDNA was then synthesized from the total RNA using a PrimeScript II Fidelity RT-PCR Kit (TaKaRa Bio Co., Kusatsu, Shiga, Japan) with an oligo dT primer. Quantitative PCR (qPCR) was then carried out using the synthesized cDNA along with the FastStart Essential DNA Green Master (Roche, Basel, Switzerland) and a Light Cycler Nano instrument (Roche, Basel, Switzerland). The quantification of *GstD1* gene expression was performed using specific primers: the sense primer, 5′-AAGATCAATCCCCAGCACAC-3′, and the antisense primer, 5′-GGTCTTGCCGTACTTCTCCA-3′). The ribosomal protein (Rp) 49, with sense primer 5′-TTCCTGGTGCACAACGTG-3′ and antisense primer 5′-TCTCCTTGCGCTTCTTGG-3′, was used as a normalized reference. The levels of target cDNAs were normalized to Rp49 expression and then calculated in terms of relative expression compared to the untreated group. The PCR protocol involved denaturing the template DNA at 95 °C for 10 min, followed by 45 cycles of 95 °C for 10 s, 60 °C for 10 s, and 72 °C for 15 s. After the final elongation reaction at 72 °C for 30 s, melting curve analysis was performed at temperatures ranging from 60 to 95 °C at a rate of 0.1 °C/s. Relative quantitative analysis was carried out using the ΔΔCq method with RP49 serving as the normalization reference [22].

### 4.8. Cell Culture

#### 4.8.1. Maturation of 3T3-L1 Adipocytes

3T3-L1 adipocytes were purchased from the Japanese Collection of Research Biosources (JCRB). The cells were cultured as preadipocytes in DMEM with L-glutamine supplemented with 10% fetal calf serum (FCS) and 1% penicillin under 5% CO_2_ at 37 °C. To instigate the differentiation of the preadipocytes into mature adipocytes, fully confluent 3T3-L1 preadipocytes were cultured in an induction medium containing 0.5 mM 3-isobutyl-1-methylxanthine (IBMX), 1 µM dexamethasone, 1 µg/mL insulin, and 10% fetal bovine serum (FBS) for 3 days. The cells were then incubated in a differentiation medium composed of 1 µg/mL insulin and 10% FBS for another 3 days. The maturation of adipocytes was completed by incubating the cells in a maturation medium with 0.5 µg/mL insulin and 10% FBS for an additional 3–6 days.

#### 4.8.2. Cell Culture of C2C12 Myoblast Cell Line

C2C12 cells were obtained from the American Type Culture Collection (ATCC). The cells were cultured in DMEM with L-glutamine supplemented with 10% FBS and 1% penicillin under 5% CO_2_ at 37 °C. Once the cells reached 80% confluency, they were harvested for further studies.

### 4.9. Cytotoxicity of Anoctochillus burmannicus Ethanolic Extract (ABE)

C2C12 cells were seeded at a density of 3 × 10^3^ cells/well (96-well), while 3T3-L1 adipocytes were seeded at a density of 4 × 10^3^ cells/well (96-well). The 3T3-L1 cells were stimulated to differentiate into mature adipocytes as mentioned above. Following this, the cells were treated with various doses of ABE (12.5–200 µg/mL) for 48 h. Cell viability was determined by sulforhodamine B (SRB) assay. The treated cells were fixed with a 10% (*w*/*v*) solution of trichloroacetic acid and stained with SRB for 30 min. Then, excess dye was removed by repeated washing of the cells with a 1% (*v*/*v*) solution of acetic acid. The protein-bound dye was dissolved in a 10 mM Tris-base solution, and the absorbance was measured at 510 nm using a microplate reader (Synergy Hybrid Reader, BioTek). The percentage of cell viability was calculated using the following equation, and the non-toxic concentrations were identified for further studies.

Cell viability (%) = (Absorbance (sample)/(Absorbance (control) × 100

### 4.10. Detection of Intracellular ROS Levels in C2C12 Myoblast

The levels of intracellular ROS in C2C12 myoblasts were investigated as described by Kim et al., with slight modifications using 2′,7′- dichlorofluorescin diacetate (DCFDA) ROS assay [86]. After entering the cells, DCFDA is deacetylated by cellular esterase, and then oxidized by ROS to produce 2′,7′–dichlorofluorescein (DCF), a highly fluorescent compound that can be detected using fluorescence spectroscopy. C2C12 myoblasts were seeded in a 24-well plate (4 × 10^4^ cells/well) and left overnight. The cells were then treated with ABE at concentrations of 50, 100, and 200 µg/mL for 24 h. The treated cells were incubated with 15 µM DCFDA in a medium without serum and phenol red for 30 min in the dark. After incubation, the cells were washed with PBS, and intracellular ROS levels were induced further by adding 200 μM H_2_O_2_ for an additional 30 min The cells were washed with PBS again and then analyzed to establish ROS levels using an inverted fluorescence microscope (ZEISS Axio Observer 7, Jena, Germany). DCF fluorescence was measured at λex = 485 and λem = 535 nm, respectively. Images were captured from five to eight fields for each treatment group, and average fluorescence pixel intensity was analyzed using Zeiss ZEN Pro software.

### 4.11. Determination of Anti-Insulin Resistance Activity by Cellular Glucose Uptake Assay

In this study, TNF-α was used as an example of an inflammatory cytokine that impairs insulin sensitivity, resulting in reduced glucose uptake in adipocytes. Mature 3T3-L1 adipocytes were treated with 2 ng/mL TNF-α in the presence of ABE (0–200 µg/mL) for 24 h. Then, the cells were collected to determine glucose uptake using the fluorescent glucose analog 2 [N-(7-nitrobenz-2-oxa-1,3-diazol-4-yl0-amono) (2-NBDG) assay [43]. The cells were incubated with low-glucose DMEM without serum for 3 h at 37 °C. Next, the cells were washed with PBS and centrifuged at 4 °C, 6000 rpm. They were then incubated with 80 µM 2-NBDG, with or without 50 nM insulin, at 37 °C for 1 h. After washing, fluorescent images of the adhered cells were captured using an inverted fluorescence microscope (ZEISS Axio Observer 7, Jena, Germany) to measure the intracellular levels of the fluorescent glucose analog. Finally, the cells were lysed with 90% DMSO, and the fluorescence intensity was measured using a microplate reader at λex = 485 and λem = 535 nm.

### 4.12. Statistical Analysis

All values are given as mean ± standard deviation (SD) from triplicate samples of three independent experiments. A survival curve was calculated following Kaplan–Meier survival estimation and analyzed using a log-rank test to compare the treatment and control groups. For the other experiments, differences between the treatment groups were assessed using a one-way analysis of variance (ANOVA). Differences between individual groups were analyzed using either a Student’s t-test or one-way ANOVA, followed by Tukey’s multiple comparisons test (GraphPad Prism version 10.4.2, GraphPad Software, Boston, MA, USA). *p*-values < 0.05 were regarded as statistically significant.

## 5. Conclusions

In conclusion, the results of this study indicate that *Anoectochilus burmannicus* ethanolic extract (*ABE*) holds promise as an anti-aging agent against oxidative stress-induced accelerated aging in this Drosophila model. ABE significantly extended adult lifespan and delayed age-related phenotypes, specifically the impairment in locomotor activity and the formation of protein aggregates in adult muscle. Moreover, ABE effectively reduced the accumulation of oxidative stress factors in muscle, as indicated by the decreased level of *Gstd1,* a *Drosophila* gene marker associated with oxidative stress, and its ability to reduce ROS production in mammalian myoblasts. These findings suggest that the underlying anti-aging mechanisms of ABE may involve enhancement of the antioxidant defense system and attenuation of inflammation-inducing insulin resistance. The evidence from this study supports the possibility that ABE has the potential to serve as a beneficial functional food or dietary supplement, particularly for middle-aged and elderly individuals, to help prevent morbidity and improve the quality of life in later years. Further studies in animal models and clinical trials are also needed to fully elucidate the therapeutic effects of ABE, as *Drosophila* models exhibit some important biological limitations in human disease research due to genetic and biochemical divergence. Moreover, the standardization of the use of the bioactive compounds from ABE and their safety information in animals and humans is essential for quality control in functional food applications.

## Data Availability

The datasets used and/or analyzed during the current study are available from the corresponding author upon reasonable request.
